# DCTPP1 attenuates the sensitivity of human gastric cancer cells to 5-fluorouracil by up-regulating MDR1 expression epigenetically

**DOI:** 10.18632/oncotarget.11864

**Published:** 2016-09-06

**Authors:** Li-liang Xia, Ya-bin Tang, Fei-fei Song, Ling Xu, Ping Ji, Shu-jun Wang, Ji-min Zhu, Yong Zhang, Guo-ping Zhao, Ying Wang, Tao-tao Liu

**Affiliations:** ^1^ State Key Laboratory of Genetic Engineering, Department of Microbiology, School of Life Sciences and Institute of Biomedical Sciences, Fudan University, Shanghai, China; ^2^ Shanghai Institute of Immunology, Department of Immunology and Microbiology, Department of Pharmacology and Chemical Biology, Shanghai Jiaotong University School of Medicine, Shanghai, China; ^3^ Department of Gastroenterology, Zhongshan Hospital, Fudan University, Shanghai, China; ^4^ Shanghai-MOST Key Laboratory of Health and Disease Genomics, Chinese National Human Genome Center at Shanghai, Shanghai, China; ^5^ Department of Microbiology and Li Ka Shing Institute of Health Sciences, The Chinese University of Hong Kong, Prince of Wales Hospital, Shatin, New Territories, Hong Kong SAR, China

**Keywords:** dCTP pyrophosphatase 1, chemoresistance to 5-fluorouracil, 5-methyl-dCTP, methylation, multidrug resistance 1

## Abstract

Gastric cancer (GC) is among the most malignant cancers with high incidence and poor prognoses worldwide as well as in China. dCTP pyrophosphatase 1 (DCTPP1) is overexpressed in GC with a poor prognosis. Given chemotherapeutic drugs share similar structures with pyrimidine nucleotides, the role of DCTPP1 in affecting the drug sensitivity in GC remains unclear and is worthy of investigation. In the present study, we reported that *DCTPP1*-knockdown GC cell line BGC-823 exhibited more sensitivity to 5-fluorouracil (5-FU), demonstrated by the retardation of cell proliferation, the increase in cell apoptosis, cell cycle arrest at S phase and more DNA damages. Multidrug resistance 1 (MDR1) expression was unexpectedly down-regulated in *DCTPP1*-knockdown BGC-823 cells together with more intracellular 5-FU accumulation. This was in large achieved by the elevated methylation in promoter region of *MDR1* gene. The intracellular 5-methyl-dCTP level increased in *DCTPP1*-knockdown BGC-823 cells as well. More significantly, the strong correlation of DCTPP1 and MDR1 expression was detectable in clinical GC samples. Our results thus imply a novel mechanism of chemoresistance mediated by the overexpression of DCTPP1 in GC. It is achieved partially through decreasing the concentration of intracellular 5-methyl-dCTP, which in turn results in promoter hypomethylation and hyper-expression of drug resistant gene *MDR1*. Our study suggests DCTPP1 as a potential indicative biomarker for the predication of chemoresistance in GC.

## INTRODUCTION

Gastric cancer (GC) is of great threat to human worldwide due to its high incidence and mortality among cancers [1, 2]. In China, GC is identified as the second leading cause of cancer death [3]. Palliative chemotherapy is critical for GC treatment since most of GC patients are initially diagnosed at an advanced stage when surgical resection is not viable [4]. However, the overall benefits of chemotherapy are limited due to drug resistance during treatment [5]. Therefore, to identify new targets involved in drug resistance may foster opportunities to develop new strategies for the improvement of chemotherapy against GC.

Nucleoside triphosphate pyrophosphatases (NTP- PPases) belong to nuclease family that are capable of hydrolyzing α-β phosphodiester bond of (d)NTPs into corresponding monophosphate and pyrophosphoric acid (PPi) [6]. To date, an array of NTP-PPases targeting non-canonical nucleotides have been identified from prokaryotes to mammalians. They are demonstrated to guarantee genome stability through preventing aberrant incorporation of non-canonical nucleotides into double-strand DNA during replication, exerting “house-cleaning” function [6, 7]. For example, MutT specifically catalyzes oxidative non-canonical nucleotides, such as 8-oxo-dGTP and 8-oxo-GTP, and prevents AT to CG mutation in *Escherichia coli* [8, 9]. MazG from *Mycobacterium tuberculosis* can safeguard genetic stability via degrading 5-OH-dCTP [10]. Certain NTP-PPases are identified in mammalians with similar biological functions, such as dUTPase [11], ITPase [12] and MTH1 [13, 14]. Moreover, dUTPase and MTH1 are reported to be associated with carcinogenesis and tumor progression [15–18], potentiating their significance in clinic [16, 19, 20].

dCTP pyrophosphatase 1 (DCTPP1) is an NTP-PPase newly identified in human whose structure contains a bacterial MazG domain [21]. It hydrolyses dCTP, 5-methyl-dCTP and 5-halo-dCTPs with specificity whereas different efficacy [21, 22]. Functional study indicates that DCTPP1 preserves genome integrity through degrading the non-canonical deoxycytidine analogues, such as 5-iodo-2′-deoxycytidine and 5-methyl-2′-deoxycytidine [22]. Our previous study showed that DCTPP1 was highly expressed in multiple carcinomas and exhibited nucleic accumulation in cancer cells, including GC [23]. What's more, high expression of DCTPP1 was strongly correlated with a poor prognosis in breast cancer [21] and GC [24]. DCTPP1 was involved in promoting cell proliferation of MCF-7 cells largely through controlling 5-methyl-dCTP metabolism and global DNA hypomethylation [21]. These results highlight the roles of DCTPP1 in cancer progression.

It is previously reported that the putative DCTPP1 inhibitors enhance the cytotoxicity against leukemia cells, including 5-azacytidine, decitabine, and gemcitabine [25]. Considering the structure similarity of chemotherapy drugs to dCTP nucleotides, the role of DCTPP1 in chemotherapy is worthy of exploration. In the present study, we investigated the effects of DCTPP1 on drug resistance to 5-FU in GC-derived cell line BGC-823 cells and further explored the underlying mechanisms.

## RESULTS

### Knockdown of *DCTPP1* increases drug sensitivity to 5-FU in BGC-823 cells

**Figure 1:**
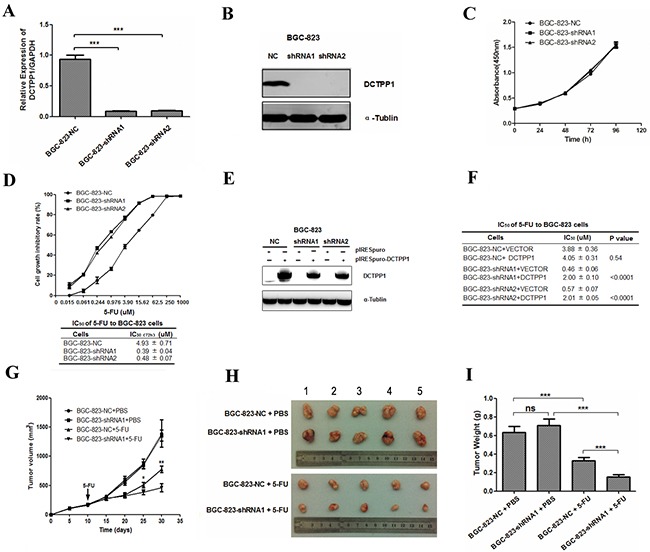
*DCTPP1* knockdown in BGC-823 cells and its effects on cell proliferation upon 5-FU treatment **A.** DCTPP1 expressions in *DCTPP1*-knockdown and control BGC-823 cells were determined by real-time PCR with *GAPDH* as an internal reference. **B.** DCTPP1 expressions in BGC-823 cells were determined by Western blot. **C.** *In vitro* cell proliferation curves of *DCTPP1*-knockdown and control BGC-823 cells detected by CCK-8 assay. **D.** Upper: cell proliferation inhibitory curves of *DCTPP1*-knockdown and control BGC-823 cells upon 5-FU treatment. Lower: IC_50_ values of *DCTPP1*-knockdown and control BGC-823 cells upon 5-FU treatment for 72 h *in vitro*. **E.** DCTPP1 expression was determined by Western blot analysis in BGC-823-NC, BGC-823-shRNA1 and BGC-823-shRNA2 cells transiently transfected with *DCTPP1* gene. **F.** IC_50_ values of *DCTPP1*-knockdown and control BGC-823 cells with rescue expression of DCTPP1 upon 5-FU treatment for 72 h. **G.** Tumor growth curves of BGC-823 cells in nude mice. Nude mice were injected subcutaneously with 3 × 10^6^ cells/mouse. When the volumes of tumors reached 180-200 mm^3^, the mice were intraperitoneally injected with 5-FU (25 mg/kg) or PBS. Tumor volumes were measured every five days for 4 weeks (n = 5). **H.** Xenograft tumors were stripped from nude mice and imaged. **I.** Tumor weights were assessed after sacrifice (n = 5 in each group). All the values shown were represented as means ± SD. (ns: not significant; *: P < 0.05; **: P < 0.01; ***: *P* < 0.001 vs control by two-tailed Student's *t*-test).

To elucidate the roles of DCTPP1 in chemoresistance, we successfully established two *DCTPP1* stable knockdown BGC-823 cells (BGC-823-shRNA1 and BGC-823-shRNA2) by transfecting vectors containing short hairpin RNA (shRNA) specific to *DCTPP1* (Table 1). DCTPP1 expression dramatically decreased at both mRNA and protein levels (Figure 1A and 1B). Although knockdown of *DCTPP1* had no impact on the proliferation of BGC-823 cells *in vitro* (Figure 1C), it increased the sensitivity of both BGC-823-shRNA1 and BGC-823-shRNA2 cells to 5-FU *in vitro* with significant decrease in IC_50(72h)_ of 5-FU when compared to BGC-823-NC cells (Figure 1D). The increased sensitivity to 5-FU induced by *DCTPP1* knockdown could be partially rescued by transient expression of *DCTPP1* in *DCTPP1*-knockdown BGC-823 cells (Figure 1E and 1F). In mouse xenograft experiments, tumor growth of BGC-823-shRNA1 was dramatically slower than that of BGC-823-NC in BALB/c nude mice after 5-FU treatment (Figure 1G to 1I). Therefore, our results indicate that knockdown of *DCTPP1* increases the sensitivity to 5-FU in BGC-823 cells both *in vitro* and *in vivo*.

**Table 1:** Oligonucleotides used in the study

Name	Sequence	Purpose
*DCTPP1* forward	5′-CGCCTCCATGCTGAGTTTG-3′	Real-time PCR
*DCTPP1* reverse	5′-CCAGGTTCCCCATCGGTTTTC-3′	
*MDR1* forward	5′-TGCGACAGGAGATAGGCTG-3′	Real-time PCR
*MDR1* reverse	5′-GCCAAAATCACAAGGGTTAGCTT-3′	
*GAPDH* forward	5′-AAGGTGAAGGTCGGAGTCAAC-3′	Real-time PCR
*GAPDH* reverse	5′-GGGGTCATTGATGGCAACAATA-3′	
*DCTPP1*-F Primer	5′-CCCGGATCCATGTCTGTGGCCGG-3′	cDNA amplication
*DCTPP1*-R Primer	5′-CCCAAGCTTCTAGGTTGAGGTCTG-3′	
*MDR1*-BSP-F	5′-TGTAACGGAAGTTAGAATATTTTTTTTGG-3′	Bisulfite sequencing PCR
*MDR1*-BSP-R	5′-AACTATCCCATAATAACTCCCAACTTTAC-3′	
shRNA1 sense	5′-GATCCGCCCTTCAAGAGGAGCTTATTCAAGA GATAAGCTCCTCTTGAAGGGCTTTTTTACGCGTG-3′	DCTPP1 Knockdown
shRNA1 antisense	5′-AATTCACGCGTAAAAAAGCCCTTCAGAGGAG CTTATCTCTTGAAGCTCCTCTTGAAGGGCG-3′	
shRNA2 sense	5′-GATCCGCCGCAAGTATACAGAATTGTTCAA GAGACAATTCTGTATACTTGCGGTTTTTTACGCGTG-3′	DCTPP1 Knockdown
shRNA2 antisense	5′-AATTCACGCGTAAAAAACCGCAAGTATACA GAATTGTCTCTTGAACAATTCTGTATACTTGCGGCG-3′	
NC-sense	5′-GATCCGTGCGTTGCTAGTACCAACTTCAAG AGATTTTTTACGCGTG-3′	Negative Control
NC-antisense	5′-AATTCCACGCGTAAAAAATCTCTTGAAGTTG GTACTAGCAACGCACG-3′	

### Knockdown of *DCTPP1* induces more apoptosis in BGC-823 cells upon 5-FU treatment

**Figure 2:**
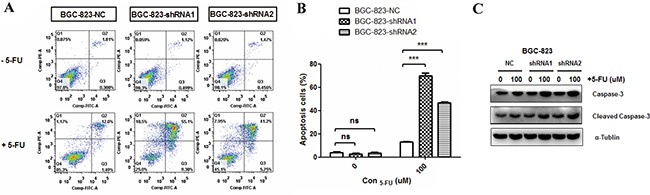
Effects of *DCTPP1* knockdown on 5-FU-induced apoptosis in BGC-823 cells **A.** Cells were treated with or without 100 μM 5-FU for 48 h and apoptosis was examined by using FITC-Annexin V/PI staining. The fluorescence intensity of FITC-Annexin V was plotted on the x-axis, and PI was plotted on the y-axis. FITC^−^/PI^−^, FITC^+^/PI^−^, FITC^+^/PI^+^, FITC^−^/PI^+^ was regarded as living, early apoptotic, late apoptotic and necrotic cells, respectively. **B.** The statistical analysis of apoptotic BGC-823 cells (FITC^+^) with or without 5-FU treatment. **C.** Caspase-3 and cleavage caspase-3 levels in *DCTPP1*-knockdown and control BGC-823 cells upon 5-FU treatment were measured by Western blot. All the values shown were represented as means ± SD. (ns: not significant; ***: *P* < 0.001 vs control by two-tailed Student's *t*-test).

Apoptosis is one of the major mechanisms responsible for cell death induced by 5-FU [26]. To investigate the effect of *DCTPP1* knockdown on apoptosis, BGC-823 cells were treated with 100 μM 5-FU for 48 h and the apoptotic cells were probed by using dual staining with PI and Annexin V (Figure 2A). The results indicated that upon 5-FU treatment the apoptotic rates of BGC-823-shRNA1 (69.67% ± 4.56%) and BGC-823-shRNA2 (46.85% ± 1.06%) cells were remarkably higher than that of BGC-823-NC cells (13.07% ± 0.72%) (*P* < 0.001) (Figure 2B). More cleavage caspased-3 was detectable in BGC-823-shRNA1 and BGC-823-shRNA2 cells (Figure 2C). These results support that knockdown of *DCTPP1* promotes the apoptosis of BGC-823 cells induced by 5-FU *in vitro*.

### Knockdown of *DCTPP1* arrests cell cycle of BGC-823 cells at S-phase after 5-FU treatment

**Figure 3:**
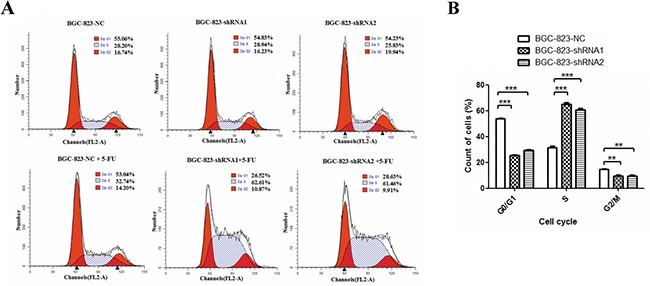
Effects of *DCTPP1* knockdown on 5-FU-induced cell cycle arrest in BGC-823 cells **A.** Cells were treated with or without 1 μM 5-FU for 48 h. Cell cycle distribution was measured by using PI staining and flow cytometry analysis. One representative flow cytometric analysis of cell cycle distribution was shown. **B.** The statistical analysis of cell cycle distribution in BGC-823 cells upon 5-FU treatment. All the values shown were represented as means ± SD. (**: *P* < 0.01; ***: *P* < 0.001 vs control by two-tailed Student's *t*-test).

Cell cycle arrest is another major mechanism of proliferation impairment in cancer cells induced by 5-FU [26]. To evaluate the effect of DCTPP1 on cell cycle arrest, we detected the cell cycle distribution of BGC-823 cells treated with or without 1 μM 5-FU for 48 h. Knockdown of *DCTPP1* alone had little effect on cell cycle arrest in BGC-823 cells, which was consistent with the results from proliferation assay (Figure 1C). However, more BGC-823-shRNA1 (65.11% ± 2.32%) and BGC-823-shRNA2 (60.85% ± 1.51%) cells were observed arresting at S-phase than BGC-823-NC cells (31.56% ± 1.73%) after 5-FU treatment (*P* < 0.001) (Figure 3A). The increase of cell population in S-phase was accompanied by a concomitant reduction in G0/G1 and G2/M phases in *DCTPP1*-knockdown BGC-823 cells (Figure 3B). These findings indicate that knockdown of *DCTPP1* leads to more S-phase arrests upon 5-FU treatment in BGC-823 cells.

### Knockdown of *DCTPP1* in BGC-823 cells increases DNA damage when treated with 5-FU

**Figure 4:**
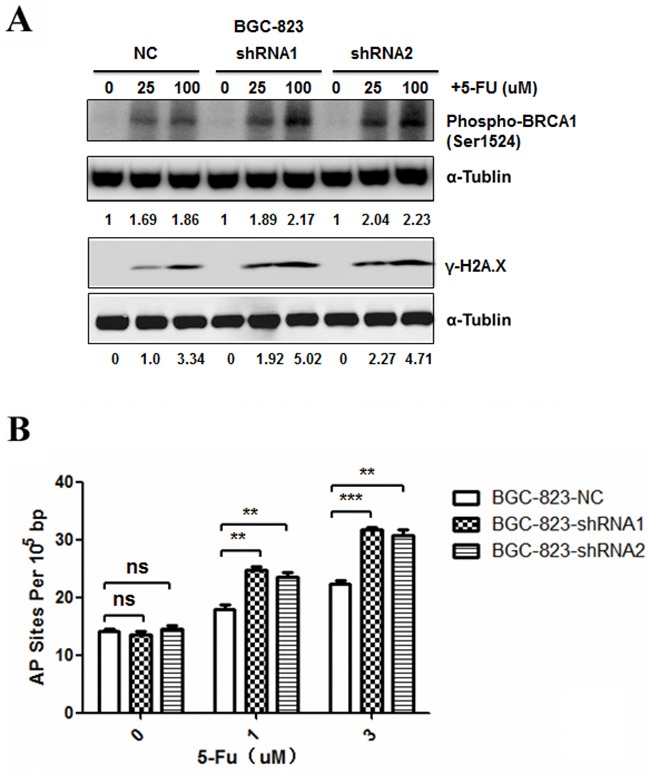
Effects of *DCTPP1* knockdown on 5-FU-induced DNA damage in BGC-823 cells **A.** DNA damage response biomarkers γ-H2AX and phospho-BRCA1 (Ser1524) were examined by Western blot in BGC-823 cells treated with 5-FU (0, 25, 100 μM) for 24 h. **B.** AP site levels were measured by DNA damage quantification kit in BGC-823 cells treated with 5-FU (0, 1, 3 μM) for 48 h. All the values shown were represented as means ± SD. (ns: not significant; **: *P* < 0.01; ***: *P* < 0.001 vs control by two-tailed Student's *t*-test).

5-FU is a fluoropyrimidine agent whose metabolites can induce DNA damage through incorporation into DNA during replication [27]. To examine the effect of DCTPP1 on DNA damage, response biomarkers for DNA damage, such as γ-H2AX and phospho-BRCA1 (Ser1524) were determined by Western blot in BGC-823 cells after 5-FU treatment for 24 h. It was found that 5-FU treatment induced more γ-H2AX and phospho-BRCA1 in BGC-823-shRNA1 and BGC-823-shRNA2 cells than in BGC-823-NC cells in a dose-dependent manner (Figure 4A). AP site is a location in DNA that has neither a purine nor a pyrimidine base due to DNA damage [28]. 5-FU treatment causes the incorporation of uracil into DNA strands, leading to the formation of AP sites during base excision repair [29]. DNA damage caused by 5-FU was further compared by counting AP sites in BGC-823 cells. Our results showed that contrary to the comparable number of AP sites between *DCTPP1*-knockdown and control BGC-823 cells without 5-FU treatment, more AP sites were detected in two *DCTPP1*-knockdown BGC-823 cells than control cells after 5-FU treatment even at low concentrations (Figure 4B). These results demonstrate that knockdown of *DCTPP1* triggers more DNA damages in BGC-823 cells as well when treated with 5-FU.

### Apoptosis and 5-FU metabolism related gene expressions have no significant changes in *DCTPP1*-knockdown BGC-823 cells

**Figure 5:**
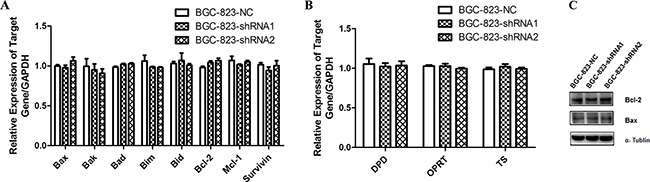
The expression of apoptosis-associated and 5-FU metabolism related genes in *DCTPP1*-knockdown and control BGC-823 cells **A.** The mRNA levels of pro-apoptotic (*Bax*, *Bak*, *Bad*, *Bim*, *Bid*) and anti-apoptotic (*Bcl-2*, *Mcl-1*, *Survivin*) genes were determined in *DCTPP1*-knockdown and control BGC-823 cells by real-time PCR. **B.** The mRNA levels of 5-FU metabolism related enzymes *DPD*, *OPRT* and *TS* in *DCTPP1*-knockdown and control BGC-823 cells were measured by real-time PCR. **C.** The protein levels of Bax and Bcl-2 in *DCTPP1*-knockdown and control BGC-823 cells were measured by Western blot.

Considering the increase of apoptosis in *DCTPP1*-knockdown BGC-823 cells induced by 5-FU, we measured the mRNA levels of apoptosis-associated genes [30, 31] by real-time PCR, including pro-apoptotic (*Bax*, *Bak*, *Bad*, *Bim* and *Bid*) and anti-apoptotic genes (*Bcl-2*, *Mcl-1* and *Survivin*) (primer sequences were listed in Supplementary Table S1). However, no significant changes were observed in apoptosis-associated genes between *DCTPP1*-knockdown and control BGC-823 cells (Figure 5A). We further chose Bax and Bcl-2 to measure their protein levels by Western blot, and found that the expressions of Bax and Bcl-2 had no significant changes after *DCTPP1* knockdown in BGC-823 cells (Figure 5C).

Drug metabolism [27] is another mechanism responsible for drug resistance. Dihydropyrimidine dehydrogenase (DPD), orotate phosphoribosyltransferase (OPRT) and thymidylate synthase (TS) are enzymes that play key roles in 5-FU metabolism as well as drug resistance in carcinoma cells [32]. But the mRNA levels of *DPD*, *OPRT* and *TS* had no change in *DCTPP1*-knockdown BGC-823 cells either (Figure 5B). These results exclude the most common two possibilities for drug resistance.

### MDR1 expression is downregulated in *DCTPP1*-knockdown BGC-823 cells

**Figure 6:**
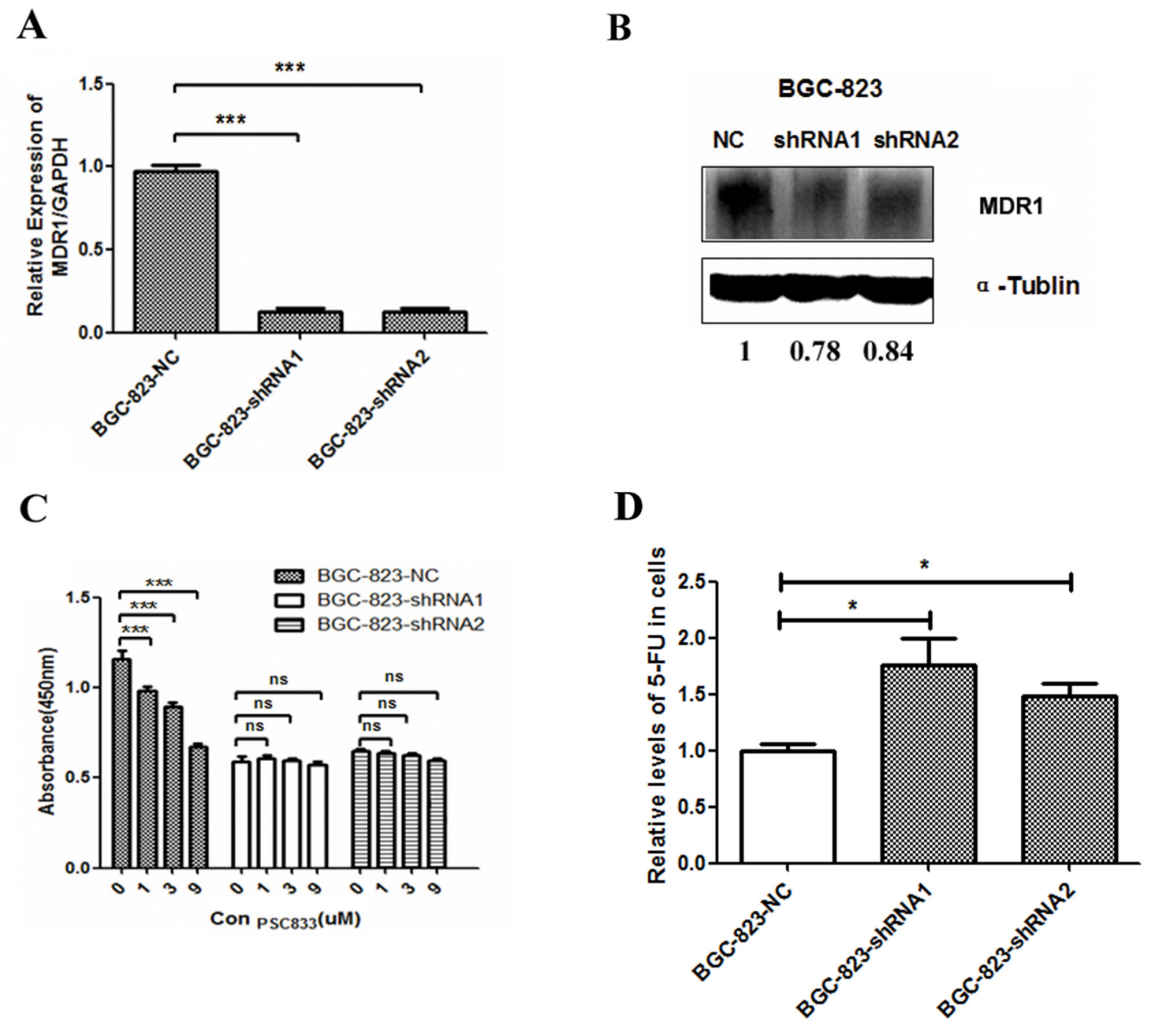
MDR1 expression in *DCTPP1*-knockdown BGC-823 cells **A.** The mRNA level of *MDR1* was measured by real-time PCR in *DCTPP1*-knockdown and control BGC-823 cells. **B.** The protein level of MDR1 was measured by Western blot in *DCTPP1*-knockdown and control BGC-823 cells. **C.** The effect of MDR1 on drug sensitivity to 5-FU in BGC-823 cells was tested by using MDR1 specific inhibitor PSC833. Cells were exposed to 2 μM 5-FU combined with PSC833 (0, 1, 3, 9 μM) for 72 h. Cell viability was detected by CCK-8 assay. **D.** The intracellular concentrations of 5-FU in *DCTPP1*-knockdown and control BGC-823 cells were measured by LC-MS/MS assay. All the values shown were represented as means ± SD. (ns: not significant; *: *P* < 0.05; ***: *P* < 0.001 vs control by two-tailed Student's *t*-test).

P-glycoprotein (P-gp) is a classical protein responsible for multi-drug resistance [33]. It is encoded by *multidrug resistance 1* (*MDR1*) and functions as an ATP-dependent drug efflux pump that reduces intracellular concentrations of chemotherapeutic agents including 5-FU [34]. Therefore, we measured the *MDR1* expression in *DCTPP1*-knockdown and control BGC-823 cells. It was shown that both the mRNA and protein levels of *MDR1* significantly decreased in *DCTPP1*-knockdown BGC-823 cells when compared to control cells (Figure 6A and 6B). PSC833, a P-gp specific inhibitor [35], was used to verify the involvement of MDR1 in the chemosensitivity to 5-FU in BGC-823 cells. PSC833 together with 5-FU treatment significantly slowed down the proliferation of BGC-823-NC cells with high MDR1 expression in a dose-dependent manner. However no effect was observed in BGC-823-shRNA cells with lower MDR1 expression (Figure 6C). Moreover, LC-MS/MS assay showed that the intracellular 5-FU levels in *DCTPP1*-knockdown BGC-823 cells were significantly higher than that in BGC-823 control cells when treated with 100 μM 5-FU for 1 h (Figure 6D). Our results thus indicate that the expression of MDR1 is downregulated in *DCTPP1*-knockdown BGC-823 cells associated with the increased intracellular 5-FU accumulation, which probably dedicates to the increased chemosensitivity to 5-FU observed in *DCTPP1*-knockdown BGC-823 cells.

### Hypermethylation of CpG islands in *MDR1* gene promoter region is obvious in *DCTPP1*-knockdown BGC-823 cells

**Figure 7:**
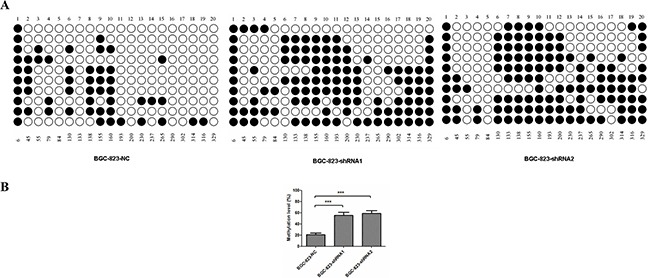
DNA methylation of *MDR1* promoter region in *DCTPP1*-knockdown and control BGC-823 cells 20 CpG dinucleotides in *MDR1* promoter region associated with MDR1 expression were selected for methylation analysis by bisulfite sequencing PCR. **A.** The methylation stature of 20 CpG dinucleotides in BGC-823-NC, BGC-823-shRNA1 and BGC-823-shRNA2 cells. “○” represented unmethylated CpG site and “•” for methylated CpG site. **B.** The statistical analysis of methylation levels in *DCTPP1*-knockdown and control BGC-823 cells. All the values shown were represented as means ± SD. (***: *P* < 0.001 vs control by two-tailed Student's *t*-test).

Epigenetic modification is one of the key mechanisms to regulate gene expression. Given the fact that down-regulation of *MDR1* is reported to be due to CpG hypermethylation in promoter region in certain cancers [36–38], we further compared the methylation in promoter region of *MDR1* gene between BGC-823-NC and BGC-823-shRNA cells. 20 CpG islands in *MDR1* gene promoter region were subjected to methylation analysis by using a bisulfite sequencing PCR approach. 10 clones for each CpG island were randomly selected for sequencing (Figure 7A). It was shown that the average methylation percentage of CpG islands in *MDR1* promoter region of BGC-823-NC cells was 26.5%. However, those in BGC-823-shRNA1 and BGC-823-shRNA2 cells were 55.5% and 59.0%, respectively, which was about 2-fold higher than in BGC-823-NC cells (Figure 7B). This was remarkably consistent with the down-expression of *MDR1* in *DCTPP1*-knockdown BGC-823 cells.

### *DCTPP1* knockdown leads to the increased intracellular concentration of 5-methyl-dCTP in BGC-823 cells

**Figure 8:**
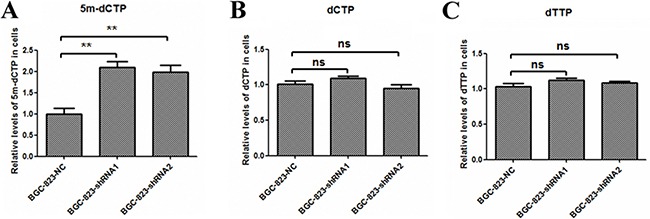
Intracellular 5-methyl-dCTP, dCTP and dTTP concentrations in *DCTPP1*-knockdown and control BGC-823 cells. The concentrations of **A.** 5-methyl-dCTP, **B.** dCTP, and **C.** dTTP in *DCTPP1*-knockdown and control BGC-823 cells were measured by LC-MS/MS assay. All the values shown were represented as means ± SD. (ns: not significant; **: *P* < 0.01 vs control by two-tailed Student's *t*-test).

DCTPP1 hydrolyzes dCTP and its derivates, such as 5-methyl-dCTP, with different efficacy [21]. It was previously reported that the high concentration of intracellular 5-methyl-dCTP increased the incorporation into DNA leading to hypermethylation and down-regulation of gene expression [39–41]. We further analyzed the effects of DCTPP1 on intracellular dCTP and 5-methyl-dCTP levels by LC-MS/MS assay. Contrary to the comparable concentrations of dCTP in *DCTPP1*-knockdown and control BGC-823 cells (Figure 8B), the intracellular 5-methyl-dCTP levels increased significantly by two folds in *DCTPP1*-knockdown BGC-823 cells when compared to control cells (Figure 8A). We also measured the intracellular dTTP level, a key intermediate during intracellular metabolism of 5-FU. There were no obvious variations of dTTP levels in two types of BGC-823 cells either (Figure 8C).

### DCTPP1 expression correlates strongly with MDR1 expression in gastric cancer tissues

**Figure 9:**
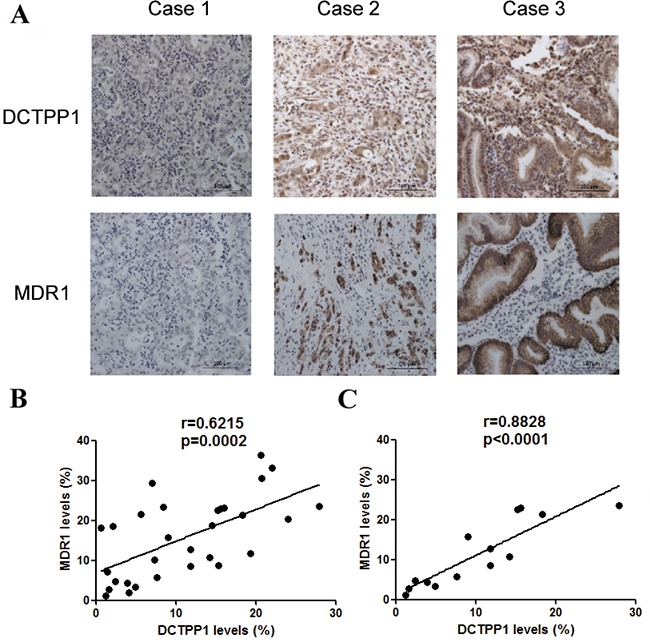
Correlation analysis of DCTPP1 and MDR1 expression in gastric cancer tissues. DCTPP1 and MDR1 expressions in GC samples were determined by immunohistochemical staining using commercial tissue microarrays **A.** The representatives of low (case 1), middle (case 2) and high (case 3) expressions of DCTPP1 and MDR1 in GC tissues were shown. **B.** The correlation between DCTPP1 expression and MDR1 expression was analyzed in GC tissues (n = 30). Each dot presented one case. **C.** The correlation between DCTPP1 expression and MDR1 expression in GC tissues with high tumor grade (II-III and III grade, n = 14). The information of pathological grades was provided by tissue microarray commercial company. The correlation was calculated by using Pearson correlation coefficient.

Since our *in vitro* study indicated that DCTPP1 potentially influenced the expression of MDR1 in BGC-823 cells, we further performed the association study between DCTPP1 and MDR1 expression in GC samples by using GC tissue microarray (Figure 9A). 30 gastric cancerous and paired adjacent regions were analyzed in parallel. It was found that the levels of DCTPP1 significantly correlated with MDR1 expression in GC tissues (r = 0.621, *P* = 0.0002) (Figure 9B), whereas no correlation was observed in adjacent tissues (r = 0.338, *P* = 0.068) (Supplementary Figure S1). Interestingly, the correlation between DCTPP1 expression and MDR1 expression was more significant in GC samples with high tumor grade (n = 14, r = 0.860, *P* < 0.0001, Figure 9C). These results thus provide clinical association of DCTPP1 and MDR1 co-expression, which partially supports the role of DCTPP1 in modulating MDR1 expression.

## DISCUSSION

DCTPP1 is the only dCTP pyrophosphatase identified in human targeting dCTP and its structure derivates. Recently, its biological function and clinical significance have been illustrated [21, 22]. In this study, we have investigated the effects of DCTPP1 on chemoresistence to 5-FU. We found that knockdown of *DCTPP1* significantly increased the sensitivity to 5-FU in GC cell line BGC-823 cells. Moreover, low expression of DCTPP1 led to the increase in intracellular 5-methyl-dCTP, which was strongly associated with the promoter hyper-methylation, leading to the subsequent low-expression of *MDR1* and the increased intracellular accumulation of 5-FU in *DCTPP1*-knockdown BGC-823 cells. These results provide new insights into the roles of DCTPP1 as a chemosensitizer in clinical application.

5-FU is one of the first-line chemotherapeutic drugs against GC. However, the overall efficacy of 5-FU treatment is somehow limited in clinic due to drug resistance [27]. The molecular mechanisms of drug resistance are complex, involving anti-apoptosis [30, 31], drug metabolism [27], and drug transportation [42]. Our results showed that the expression of apoptosis-associated and 5-FU metabolism related genes had no significant changes between *DCTPP1*-knockdown and control BGC-823 cells. These results impel us to focus on another conventional mechanism of drug resistance mediated by drug transportation.

The abnormal expressions of drug transporters, such as P-gp, multi-drug resistance protein 1 (MRP1) and mitoxantrone resistance protein (MXR/BCRP), are the major cause for chemotherapy failure [43]. Our results showed that *MRP1* and *MXR/BCRP* had no significant changes between *DCTPP1*-knockdown and control BGC-823 cells (Supplementary Figure S2). But the expression of P-gp was downregulated in *DCTPP1*-knockdown BGC-823 cells (Figure 6A and 6B). P-gp is a well-documented drug transporter. The increased expression of P-gp in GC has been reported to be associated with the poor prognosis and multidrug resistance [44, 45]. P-gp is also reported to be involved in the drug resistance to 5-FU in BGC-823 cells [46, 47]. In this study, we illustrated that P-gp expression was downregulated in *DCTPP1*-knockdown BGC-823 cells with more 5-FU accumulating in the cells (Figure 6D). This led to more cell apoptosis, cell cycle arrest as well as more DNA damage in *DCTPP1*-knockdown BGC-823 cells observed in our study.

How *DCTPP1* knockdown affects *MDR1* expression remains ambiguous. In some carcinomas, the CpG hypermethylation of *MDR1* promoter is thought to be one of the mechanisms causing down-regulation of *MDR1* [36, 48, 49]. Consistent with these findings, our results showed that the methylated CpG sites in *MDR1* promoter region significantly increased after *DCTPP1* knockdown. It is well documented that DNA methylation is an enzyme-mediated post-replication process where DNA methyltransferase (DNMT) plays a critical role [50]. We compared the expression of DNMT family members between *DCTPP1*-knockdown and control BGC-823 cells. But the expressions of *DNMT1*, *DNMT3A* and *DNMT3B* had no significant difference before and after *DCTPP1* knockdown in BGC-823 cells (Supplementary Figure S3), suggesting that other mechanism should be involved in regulating CpG hypermethylation in *MDR1* promoter region with DCTPP1 silencing.

5-methyl-dCTP is recognized as the fifth nucleotide in eukaryotic biology. It is reported previously that 5-methyl-dCTP is a nucleotide substrate of DNA polymerase and could be incorporated into DNA during replication [51]. What's more, the incorporation of 5-methyl-dCTP during DNA replication has been associated with DNA hypermethylation and gene silencing [39–41]. Therefore, the involvement of 5-methyl-dCTP in epigenetic modification highlights its significance in epigenetic inheritance. Our previous study showed that DCTPP1 could catalyze 5-methyl-dCTP preferentially *in vitro*. The intracellular 5-methyl-dCTP concentration increased in *DCTPP1*-knockdown breast cancer cell MCF-7, suggesting 5-methyl-dCTP is one of the main substrates of DCTPP1 in cells [21]. Requena et al. have also revealed that *DCTPP1*-knockdown cells were hypersensitive to 5-methyl-2′-deoxycytidine, whose triphosphate form is 5-methyl-dCTP. Addition of 5-methyl-2′-deoxycytidine induced more increment in global DNA methylation in *DCTPP1*-knockdown cells than in control cells, supporting the function of DCTPP1 on down-regulating DNA methylation though preventing the incorporation of 5-methyl-dCTP into DNA strands [22]. In the present study, we also verified the increase of 5-methyl-dCTP in *DCTPP1*-knockdown BGC-823 cells, which is consistent with our previous study [21]. The increment of CpG methylation in *MDR1* promoter region was observed as well, which supports the function of DCTPP1 on DNA methylation through modulating the intracellular 5-methyl-dCTP concentration.

DCTPP1 is overexpressed in multiple cancers including GC. Its overexpression is correlated with a poor prognosis in GC [24], making it a potential diagnostic and therapeutic target in cancer therapy. Based on our findings from tissue microarrays, we defined the strong correlation between DCTPP1 and MDR1 expression, implying the involvement of DCTPP1 in drug sensitivity through modulating MDR1 expression during chemotherapy. Accordingly, we speculate that the intensive DCTPP1 expression may be a good biomarker for predicting the responses to certain chemotherapeutics, such as 5-FU. The association between DCTPP1 expression and clinical outcome of 5-FU treatment will be further evaluated in the future, which will provide direct evidence to support the roles of DCTPP1 in the enhancement of chemotherapy efficacy. Moreover, to develop DCTPP1 inhibitors may promote the efficacy of 5-FU in the treatment of certain GC patients.

In summary, we report here the engagement of DCTPP1 in increasing the chemoresistance of BGC-823 cells to 5-FU. This largely owes to the hypomethylation of *MDR1* promoter region and the subsequent hyper-expression of protein, which in turn accelerates the efflux of intracellular 5-FU. DCTPP1 might be putatively a novel predicative biomarker for chemotherapy in the future.

## MATERIALS AND METHODS

### Cell lines and reagents

The GC cell line BGC-823 was purchased from the Shanghai Institute for Biological Sciences Chinese Academy of Sciences (Shanghai, China) and routinely maintained in DMEM medium (Gibco, Carlsbad, CA, USA) supplemented with 10% fetal bovine serum (FBS) (Gibco) in a 5% CO_2_ humidified atmosphere at 37°C.

Rabbit anti-human DCTPP1 polyclonal antibody (pAb) (Cat#AP2821a) was obtained from Abgent (San Diego, USA). Mouse anti-human MDR1 (Cat# sc-55510) and mouse anti-DNMT1 monoclonal antibody (mAb) (Cat# sc-271729) were purchased from Santa Cruz Biotechnology (Santa Cruz, CA, USA). Mouse anti-α-tublin Ab (Cat# T6074) was purchased from Sigma (Louis, MO, USA). Rabbit anti-Phospho-BRCA1 Ab (Cat# 9009), rabbit anti-Phospho-H2A.X Ab (Cat# 2197), rabbit anti-caspase-3 (8G10) mAb (Cat# 9665), rabbit anti-cleaved caspase-3 (Asp 175) mAb (Cat# 9664), rabbit anti-Bax pAb (Cat#2772), rabbit anti-Bcl-2 (50E3) mAb (Cat# 2870), HRP-linked anti-rabbit IgG Ab (Cat# 7074) and HRP-linked anti-mouse IgG Ab (Cat# 7076) were purchased from Cell Signaling Technology (Beverly, MA, USA). dCTP, dTTP and 5-FU were purchased from Sigma and 5-methyl-dCTP was purchased from New England BioLabs (Ipswich, MA, USA).

### Construction of *DCTPP1*-knockdown BGC-823 cells

Stable *DCTPP1*-knockdown BGC-823 cells were constructed by transfecting RNAi-Ready pSIREN-RetroQ retroviral vector (Clontech, CA, USA) containing shRNA oligonucleotides targeting *DCTPP1* as described in our previous report [21]. Briefly, BGC-823 cells were co-cultured with viral particles containing pSIREN-RetroQ-shRNA1, pSIREN-RetroQ-shRNA2 or pSIREN-RetroQ-negative control (NC) for 24 h (sequences of shRNA targeting *DCTPP1* and NC sequences were listed in Table 1). Complete DMEM medium containing 0.4 μg/mL puromycin (Gibco) was replaced and cells were maintained for 1 week. Stably transfected cells were cultivated in complete DMEM medium with 0.2 μg/mL puromycin for at least 1 week before proceeding for further study. The efficiency of RNA interference was determined by real-time PCR and Western blot analysis.

To rescue DCTPP1 in *DCTPP1*-knockdown BGC-823 cells, *DCTPP1* gene was amplified by PCR with the template from Hela cDNA (primer sequences were listed in Table 1) and inserted into pIRESpuro plasmid (Invitrogen) at the EcoRI and NotI sites. BGC-823 cells (4 × 10^5^/2 mL) were seeded in 6-well plates and transfected with recombinant plasmid pIRESpuro-DCTPP1 or empty vector using Lipofectamine 2000 (Invitrogen) according to the manufacturer's instructions. BGC-823 cells were trypsinized, counted and reseeded in 96-well plates for another 24 h after transfection. Cells were cultured with 5-FU (Sigma) at different concentrations (1000, 250, 62.5, 15.625, 3.906, 0.977, 0.244, 0.061, 0.015, 0 μM) for 72 h and subjected to cell viability assay.

### Cell viability assay *in vitro*

Cells (3 × 10^3^/100 μL) were seeded in 96-well plates (Corning, Steuben County, New York, USA). After 24 h, cells were cultured with 5-FU (Sigma) at different concentrations (1000, 250, 62.5, 15.625, 3.906, 0.977, 0.244, 0.061, 0.015, 0 μM) for 72 h. Cell viability was analyzed afterwards by using CCK-8 kit (Dojindo Laboratories, Kumamoto, Japan) according to the manufacturer's instructions. The absorbance was measured at 450 nm on a microplate reader (BioRad, Hercules, CA, USA). IC_50 (72h)_ value was calculated by GraphPad Prism 5 software (San Diego, CA, USA).

To investigate the roles of MDR1 in the chemosensitivity to 5-FU, BGC-823 cells were seeded in 96-well plates and treated with 5-FU and PSC833 (Medchemexpress, NJ, USA) for 72 hours. Cell viability was analyzed by using CCK-8 assay.

### Xenograft tumor growth in BALB/c-nu mice

Six-week-old BALB/c nude mice (purchased from SLAC Company, Shanghai, China) were subcutaneously injected with 3 × 10^6^ BGC-823-NC or BGC-823-shRNA1 cells. When the volumes of xenograft tumors reached 180-200 mm^3^, mice were randomly subdivided into two groups with 5 mice in each group. Experimental groups were treated with 5-FU intraperitoneally (25 mg/kg) at every other day for two weeks while control groups were treated with PBS. The volumes of xenograft tumors were measured by caliper and calculated by the following formula V = 1/2 × (length × width^2^). After 4 weeks, nude mice were sacrificed and xenograft tumors were harvested and weighed. The protocols of animal experiments were approved by the Animal Ethics Committee of SJTUSM, and were performed under the Guide for the Care and Use of Laboratory Animals.

### Apoptosis analysis

Cells (1 × 10^5^/2 mL) were seeded in 6-well plates and treated with or without 100 μM 5-FU. After incubation for 48 h, total cells were harvested and washed twice with cold PBS. Cell apoptosis was analyzed by using FITC-Annexin V Apoptosis Detection Kit (Biolegend, San Diego, CA, USA) according to the manufacturer's instructions. Cells were acquired by FACS Calibur flow cytometer (BD Pharmingen, San Diego, CA, USA) and data analysis was performed by using FlowJo 7.6 software (Tree star, Ashland, Oregon, USA).

### Cell cycle analysis

Cells (1 × 10^5^/2 mL) were seeded in 6-well plates and treated with or without 1 μM 5-FU. After incubation for 48 h, adherent cells were trypsinized, collected and washed twice with cold PBS. Cell pellets were resuspended in 5 mL ice-cold PBS containing 75% ethanol and fixed at -20°C overnight. Cells were stained with PI/RNase staining buffer (BD Pharmingen, San Diego, CA, USA) according to DNA staining protocol for flow cytometry. Cells was acquired by FACS Calibur flow cytometer (BD Pharmingen) and cell cycle was analyzed by ModFit software (Verity Software House, Topsham, ME, USA).

### DNA damage assay

Cells (1 × 10^5^/2 mL) were seeded in 6-well plates and treated with or without 5-FU. After incubation for 48 h, adherent cells were collected. Proteins from some of the cells were extracted by RIPA lysis buffer. DNA damage markers, phospho-BRCA1 and phospho-H2A.X, were measured by Western blot. In addition, chromosomal DNA was extracted from part of the cultured cells. AP sites were measured using DNA Damage Quantification kit (Dojindo Laboratories) according to the manufacturer's instructions.

### Western blot

Cells were washed twice with cold PBS, collected and lysed by RIPA lysis buffer containing 1mM phenylmethanesulfonyl fluoride (PSFM) (Beyotime, Nantong, Jiangsu, China). The whole cell extracts were collected and protein concentration was determined by BCA protein assay kit (BioRad, Hercules, CA, USA). Cell lysates were separated by 10% sodium dodecyl sulfate polyacrylamide gel electrophoresis (SDS-PAGE) and transferred to polyvinylidene difluoride (PVDF) membranes (Millipore Co., Billerica, MA, USA). The membranes were blocked with PBS (pH7.4) containing 5% non-fat milk for 2 h at room temperature and then incubated with primary antibodies overnight at 4°C. After incubation with HRP-conjugated secondary antibodies for 1 h, immunoreactive proteins were visualized with ECL detection system (Millipore Co.). Intensities of the bands were quantified by using an image analysis system (BioRad).

### Real-time PCR

Total RNA was extracted with Trizol reagent (Invitrogen Life Technologies, Carlsbad, CA, USA) according to the manufacturer's instructions. First-strand cDNA was synthesized by using a PrimeScript™ RT reagent Kit with gDNA Eraser (Takara Biotechnology, Dalian, China). The primer sequences used in real-time PCR were listed in Table 1 and Supplementary Table S1. Real-time PCR was performed using applied biosystems 7300 real-time PCR system with SYBR^®^ Premix Ex Taq™ kits (Takara Biotechnology, Dalian, China) according to the manufacturer's instructions.

### Methylation analysis

Total DNA was extracted from cultured cells using TaKaRa MiniBEST Universal Genomic DNA Extraction Kit (Takara Biotechnology). Bisulfite sequencing PCR (BSP) was performed in BioSun Biotech Co. Ltd. (Shanghai, China). Briefly, MDR1-BSP-F and MDR1-BSP-R primers were designed for the amplification of the promoter region of *MDR1* gene including 20 CpG dinucleotides that have been reported to be highly linked to the regulation of *MDR1* expression [36–38]. The BSP was carried out in 50 μL reaction system containing 0.2 mM dNTPs, 2 mM MgCl_2_, 0.2 μM primer, 12.5 units TaqHS (Takara Biotechnology), and 600 ng bisulfite-modified DNA. The BSP conditions were 94°C for 1 min, followed by 37 cycles at 94°C for 15 s, 54°C for 20 s, and 72°C for 25 s, with a final extension at 72°C for 10 min. PCR products were purified and cloned into the pEASY-T1 vector for sequencing. For each PCR sample, 10 individual clones were randomly selected and sequenced in the experiments. In the BSP assay, the treatment of bisulfite led to the conversion of cytosine residues into uracil whereas 5-methyl-cytosine residues unaffected. After PCR amplification, original unmethylated cytosines were converted to thymine while methylated cytosines remained. Therefore, methylation level was determined according to the average methylation rate of 20 CpG dinucleotides for each DNA sample.

### Liquid chromatography tandem mass spectrometry (LC-MS/MS) assay

Intracellular nucleotides were measured by LC-MS/MS as described in our previous study [21] with minor modifications. Briefly, cells were harvested and washed twice with cold saline. 5 × 10^6^ cells were extracted with 500 μL pre-cold 60% methanol and immediately frozen in liquid nitrogen. After storage at −80°C for at least 24 h, sample solutions were centrifuged at 14,000 *g* for 10 min at 4°C. The supernatants were collected and stored at −80°C until for LC-MS/MS analysis.

Intracellular 5-FU was measured by LC-MS/MS as described in previous report [52] with minor modifications. Briefly, 1.5 × 10^6^ BGC-823 cells were seeded in 10 cm dish overnight and treated with 10 mL DMEM medium containing 100 μM 5-FU. After incubation for 1 h, the medium was replaced with 10 mL DMEM medium. After incubation for another 15 min, the intracellular 5-FU was extracted by pre-cold 60% methanol as described above.

LC-MS/MS assay was performed using an Agilent 1200 HPLC system coupled to an Agilent 6410 triple quadruple mass spectrometer (Agilent Technologies, Palo Alto, CA, USA). The mode of multiple reaction monitoring (MRM) was used to identify and quantify dCTP (transition: m/z 468.1 [M + H] + → 112.1, fragmentor 120 V, collision energy 20 eV), 5-methyl-dCTP (transition: m/z 482.1 [M + H] + → 126.1, fragmentor 140 V, collision energy 20 eV), dTTP (transition: m/z 480.1 [M + H] + → 159.1, fragmentor 125 V, collision energy 20 eV), and 5-FU (transition: m/z 129 [M + H] + → 42.2, declustering potential -55 V, collision energy -25 eV). The data of peak areas were acquired and the relative quality of compound tested in BGC-823 cells was analyzed accordingly to their areas of peaks.

### Gastric cancer tissue microarray

Tissue microarray containing 30 formalin-fixed, paraffin-embedded GC tissues and 30 paired adjacent tissues (2.0 mm in diameter and 4 μm in thickness) was purchased from the Shanghai Outdo Biotech Company (Shanghai, China). The expressions of DCTPP1 and MDR1 in GC tissue microarray were tested by immunohistochemistry described in our previous report using anti-DCTPP1 and anti-MDR1 antibodies [21]. Slides were observed under the light microscope and data were analyzed using the Leica Qwin V3 image analysis system (Leica Microsystems GmbH, Wetzlar, Germany). The extent of specific staining was calculated according to the percentage of positive area to the total area of campus visualis (×100).

### Statistics analysis

All the data were represented as the means ± standard deviation (SD). Statistical analysis was carried out by using two-tailed Student's *t*-test with SPSS version 20.0 (SPSS Inc, Chicago, IL, USA). Differences between groups were determined as statistically significant at *P* < 0.05. The correlation between DCTPP1 and MDR1 expression was assessed using Pearson correlation coefficient. *P* < 0.05 was considered significant.

## SUPPLEMENTARY MATERIALS FIGURES AND TABLE


