# Supplementary material for: DCTPP1 attenuates the sensitivity of human gastric cancer cells to 5-fluorouracil by up-regulating MDR1 expression epigenetically
Source: Oncotarget. 2016 Sep 6;7(42):68623–37. doi: 10.18632/oncotarget.11864 (PMC5356578; doi:10.18632/oncotarget.11864)
Supplement: Supplementary file 1 [file oncotarget-07-68623-s001.pdf]

DCTPP1 attenuates the sensitivity of human gastric cancer cells to 5-fluorouracil by up-regulating MDR1 expression epigenetically

SUPPLEMENTARY FIGURES AND TABLE

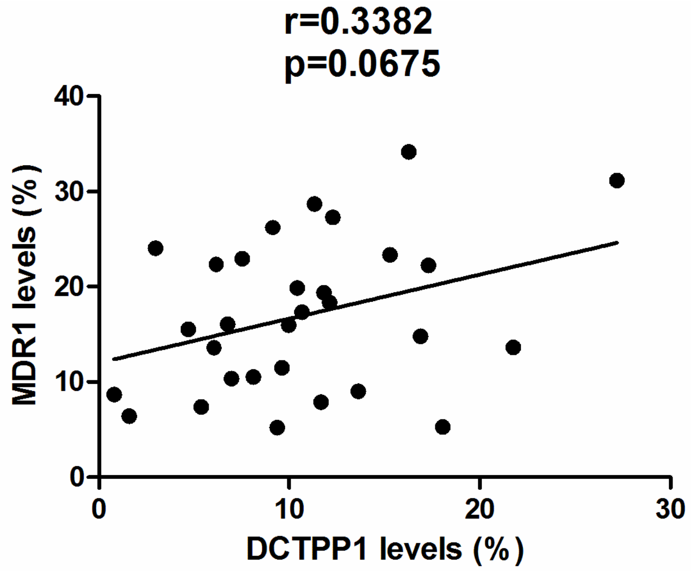

Supplementary Figure S1: The correlation analysis of DCTPP1 and MDR1 expression in gastric cancer adjacent tissues. Immunohistochemical staining was performed on gastric cancer tissue microarrays for the detection of DCTPP1 and MDR1 expressions. The correlation between DCTPP1 expression and MDR1 expression was assessed in gastric cancer adjacent tissues (n = 30) by using Pearson correlation coefficient.

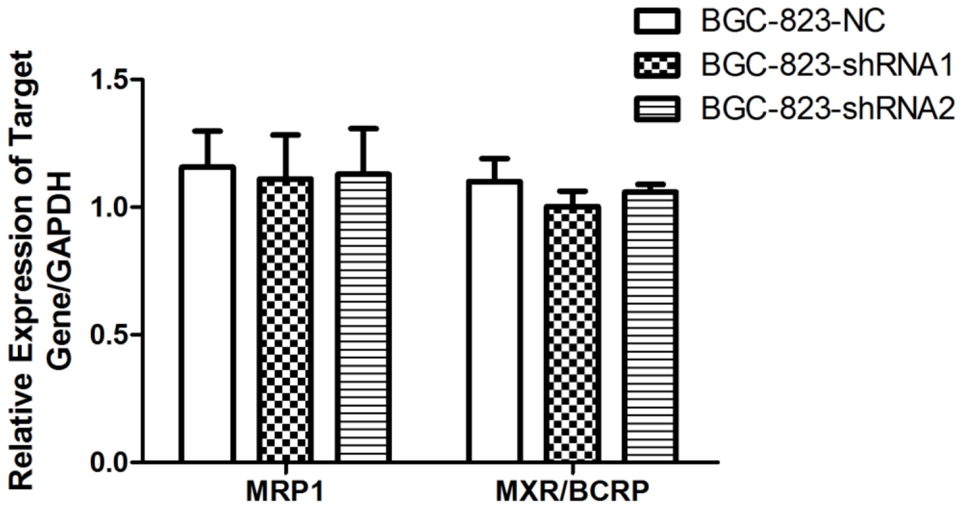

Supplementary Figure S2: Real-time PCR assay for *MRP1* and *MXR/BCRP* in *DCTPP1*-knockdown and control BGC-823 cells.

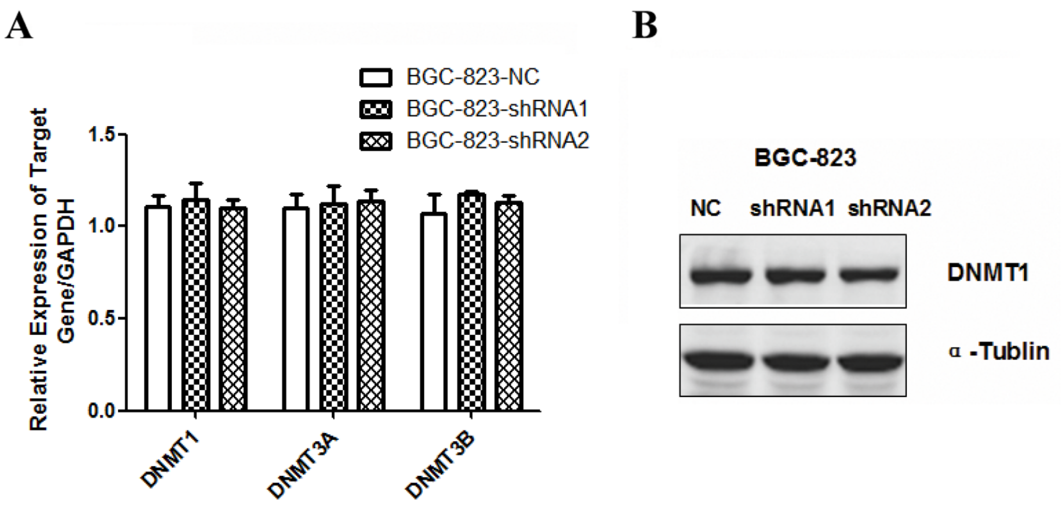

Supplementary Figure S3: Comparison of DNA methyltransferase expression in *DCTPP*1-knockdown and control BGC-823 cells. A. The mRNA levels of *DNMT1*, *DNMT3A* and *DNMT3B* in BGC-823 cells were measured by real-time PCR. **B.** Western blot analysis of DNMT1 expression in BGC-823 cells.

Supplementary Table S1: Primers used in real-time PCR

Name	Sequence
GAPDH-forward	5′-AAGGTGAAGGTCGGAGTCAAC-3′
GAPDH-reverse	5′-GGGGTCATTGATGGCAACAATA-3′
Bax-forward	5′-GGTTGTCGCCCTTTTCTACTTT-3′
Bax-reverse	5′-GTGAGGAGGCTTGAGGAGTCT-3′
Bak-forward	5′-CAGGGAAAAGGAGTAGGGAGTT-3′
Bak-reverse	5′-CCAATAGAGAAGGCACTGTCAC-3′
Bim-forward	5′-GCCACTACCACCACTTGATTCT-3′
Bim-reverse	5′-TACCTTCTCGGTCACACTCAGA-3′
Bid-forward	5′-TGAGGAAAGCAAGAATCAGAGA-3′
Bid-reverse	5′-CTCCAACAAAGGAAACGAAGTT-3′
Bcl-2-forward	5′-TCCCATCAAAACTCCTGTCTTT-3′
Bcl-2-reverse	5′-TTTCCATCCGTCTGCTCTTC-3′
Bad-forward	5′-GAGGATGAGTGACGAGTTTGTG-3′
Bad-reverse	5′-GGTGGAGTTTCGGGATGTG-3′
Mcl-1-forward	5′-CATTTCTTTTGGTGCCTTTGTG-3′
Mcl-1-reverse	5′-CCAGTCCCGTTTTGTCCTTAC-3′
Survivin-forward	5′-TGTGATGAGAGAATGGAGACAGA-3′
Survivin-reverse	5′-AGCAGTGGCAAAAGGAGTATCT-3′
DPD-forward	5′-AATGATTCGAAGAGCTTTTGAAGC-3′
DPD-reverse	5′-GTTCCCCGGATGATTCTGG-3′
TS-forward	5′-GAATCACATCGAGCCACTGAAA-3′
TS-reverse	5′-CAGCCCAACCCCTAAAGACTGA-3′
OPRT-forward	5′-TCCTGGGCAGATCTAGTAAATGC-3′
OPRT-reverse	5′-TGCTCCTCAGCCATTCTAACC-3′
MRP1-forward	5′-ATGGACTACACAAG GGTGATCG-3′
MRP1-reverse	5′-TCCGCATCTCTGT CTCTCC-3′
MXR-forward	5′-TGCAACATGTACTGGCGAAGA-3′
MXR-reverse	5′-TCTTCCACAAGCCCCAGG-3′
DNMT1-forward	5′-AGGCGGCTCAAAGATTTGGAA-3′
DNMT1-reverse	5′-GCAGAAATTCGTGCAAGAGATTC-3′
DNMT3A-forward	5′-CAATGACCTCTCCATCGTCAAC-3′
DNMT3A-reverse	5′-CATGCAGGAGGCGGTAGAA-3′
DNMT3B-forward	5′-CCATGAAGGTTGGCGACAA-3′
DNMT3B-reverse	5′-TGGCATCAATCATCACTGGATT-3′
